# Study curing of epoxy resin by Isophoronediamine/ Triethylenetetramine and reinforced with montmorillonite and effect on compressive strength

**DOI:** 10.1186/s13065-024-01319-8

**Published:** 2024-10-28

**Authors:** Soliman Mehawed Abdellatif Soliman, Mohab Abdelhakim, Magdy Wadid Sabaa

**Affiliations:** Chemistry Department, Faculty of Science, Cairo University, Giza, 12613 Egypt

**Keywords:** Diglycidyl ether of bisphenol-A, Epoxy, Triethylenetetramine, Isophoronediamine, Compressive strength, Organically modified montmorillonite

## Abstract

Epoxy is a widely used thermosetting resin recognized for its exceptional performance in adhesives, coatings, and various other applications, attributed to its high tensile strength, stiffness, electrical performance, and chemical resistance. Epoxy-clay nanocomposites are extensively employed across diverse industries. The physical and chemical properties of these nanocomposites are influenced by the processing methods, clay modifiers, and curing agents used during their preparation. In this study, epoxy/nanoclay composites based on Diglycidyl Ether Bisphenol-A (DGEBA) will be cross-linked using Isophorone Diamine (IPD), a cycloaliphatic amine, and Triethylenetetramine (TETA), a linear aliphatic amine. The initial phase of the research will assess the impact of different types of cross-linkers, both individually and in combination at various molar ratios (such as Isophorone Diamine: Triethylenetetramine (IPA: TETA) / 25:75 and 75:25), on the compressive strength of the epoxy mortar. In the subsequent phase, the epoxy formulation with an Isophorone Diamine: Triethylenetetramine (IPD: TETA / 75:25), which demonstrates the highest compressive strength, will be selected for further investigation. This formulation will be used to evaluate the effects of different weight percentages (3%, 5%, and 7%) of organically modified montmorillonite (OMMT). The prepared epoxy composites will be characterized using a range of techniques, including Fourier Transform Infrared Spectroscopy (FT-IR), Transmission Electron Microscopy (TEM), and Scanning Electron Microscopy (SEM). The epoxy/nanoclay composite with an IPD: TETA / 75:25 and 3 wt % OMMT is expected to show the highest compressive strength, which is 94 MPa.

## Introduction

Epoxy is a reactive polymer with epoxide groups, and its types vary depending on the initial compounds used, such as phenol or polyol. Bisphenol-A epoxy resin, made by reacting bisphenol-A with epichlorohydrin, is a common example. This resin cures without producing by-products, which prevents mold-related issues. Epoxy-clay nanocomposites are increasingly utilized across various industries, including aerospace, defense, and automotive sectors [1, 2]. The last decade has shown show a significant increase in research focused on epoxy nanocomposites. Studies highlighted improvements in toughness, thermal stability, and barrier properties through the addition of various nanoparticles such as clays, silica, and carbon nanotubes [3]. Research continues to explore new types of nanofillers, including graphene, carbon nanotubes, and functionalized silica, which provide enhanced mechanical and thermal properties [4]. Recent studies have demonstrated significant improvements in tensile strength, impact resistance, thermal stability, and flame retardancy in epoxy nanocomposites. The incorporation of nanoparticles often results in synergistic effects that enhance multiple properties simultaneously [5]. The properties of these epoxy systems are affected by the processing methods, clay modifiers, and curing agents used in their production. Additionally, the morphology of the clay within the nanocomposites—such as whether it is intercalated or exfoliated—is influenced by these factors [6]. In a polymer matrix, clay particles can form three types of composites: (1) Conventional phase-separated composites with poor mechanical properties due to immiscibility of polymer and clay; (2) Intercalated nanocomposites where polymer chains are inserted into clay layers; and (3) Exfoliated nanocomposites where clay layers are individually dispersed in the polymer matrix, offering improved properties due to better dispersion and increased interaction between the polymer and clay [7]. Polymer/layered-silicate nanocomposites are hybrid materials that significantly enhance the performance characteristics of a polymer through the integration of organic and inorganic components [8]. Moreover, there are different parameters affect the properties of epoxy as types of curing agent [9], amount of nanoclay and epoxy used. Various studies were done on epoxy/nanoclay composites based on diglycidyl ether bisphenol - A (DGEBA) [10–13]. Organically modified montmorillonite (O-MMT) is the most commonly used nanoclay due to its ability to readily expand through the interlayer distance (d-spacing) of the epoxy [14–16]. The chemical and corrosion resistance of epoxy is good, but it has rigid and brittle structure [17]. To improve its impact properties, nano- O-MMT is incorporated epoxy resin cross-linked. There are a large number of researches on epoxy/clay composites with different curing agents are done [18–21]. Conversely, organometallic compounds play a crucial role in developing sensitive molecular probes for detecting anionic, acidic, and metal ion contaminants [22]. Furthermore, the introduction of organosilanes into graphene oxide has enabled the electrochemical detection of uric acid [23]. In literature, there are different studies about curing Diglycidyl ether of bisphenol-A (epoxy) using Isophoronediamine without [24] and with [25] wt% of organically modified montmorillonite. The previous studies show that incorporating of organically modified montmorillonite (O-MMT) clay with epoxy improves the compressive strength about 40% [25]. The aim of the current research is to improve the compressive strength of epoxy nanocomposites. This study uniquely examines the effect of varying the stoichiometric ratios of aliphatic cyclic amine (Isophorondiamine, IPD), a linear aliphatic amine (Triethylenetetramine, TETA) on the compressive strength of cured epoxy. Specifically, we will investigate the following combinations: (1) Isophorondiamine, (2) Triethylenetetramine, and mixed cross-linkers at molar ratios of (3) IPD: TETA / 25:75 and (4) IPD: TETA / 75:25. After determining the most effective cross-linker that yields the highest compressive strength for the cured epoxy resin, we will proceed to explore how different weight percentages of organic modified montmorillonite (O-MMT) affect the compressive strength of the cured epoxy.

## Experimental

### Materials

Diglycidyl ether of bisphenol-A (DGEBA) (with a trade name of (D.E.R. ™ 331™ Liquid Epoxy Resin)) was obtained DOW Chemicals Company. Isophoronediamine (IPD) was purchased from Huntsman Company, Germany, (trade name of ARADURE 42 BD with [AHEW] of 42.6 (g.eq^− 1^). Organically modified montmorillonite (O-MMT) clay (NANOMER I.31 PS) and Triethylenetetramine were obtained from Sigma-Aldrich.

### Characterization techniques

Fourier-transform infrared spectra of epoxy composites have been recorded on Testcan Shimadzu Infrared-Spectrophotometer (model 8000) on the range of wavenumber from 4000 to 600 cm^− 1^ (25 ºC). Thermal behavior of prepared epoxy composites were tested using TGA-50 H Shimadzu Thermogravimetric Analyzer. The temperature measurements were conducted over a range of 0 to 600 ºC using a platinum pan with a heating rate of 10 ºC/min under a nitrogen atmosphere (with flow rate of 25 ml.min^− 1^). Scanning Electron Microscope (SEM) images of epoxy composites were recorded by model Quanta 250 FEG (Field Emission Gun) attached with EDX Unit (Energy Dispersive X-ray Analyses), with accelerating voltage 30 kV, magnification14x up to 1000000 and resolution for Gun.1n). The gel time of different epoxy resins was tested manually, based on the estimation of the resin’s rheological behavior [26]. Finally, the compressive strength values of the epoxy composites were measured using a MATEST Srl BREMBATE SOPRA 24030 machine (Model No. C038, Grade A) with a capacity of 1500 kN according to ASTM 579.01 method B.

### Experimental techniques

Diglycidyl ether of bisphenol-A (DGEBA) resin is cured at temperature 80 ºC. Resins are degassed under vacuum until no bubbles and followed by mixing stoichiometric ratios of isophoronediamine (IPD) and/or Triethylenetetramine (TETA) at room temperature. Uncured epoxy resins are degassed and casted into steel molds pre-treated with mold-releasing agent. Resins are stored at room temperature for 7 days for curing, then removed from the molds and post cured for 2 h at 100 ºC then 2 h at 160 ºC. For the preparation of cured resin reinforced with montmorillonite, different amounts of organically modified montmorillonite (O-MMT) are dispersed at 80 ºC for 2 h using a high-shear mechanical stirrer before adding the cross-linkers.

## Results and discussion

In the first section, mixing ratio (such as IPA: TETA / 25:75 and 75:25) between curing agents Isophoronrdiamine (IPD) and Triethylenetetramine (TETA) exhibits the highest value of epoxy’s compressive strength value. In the second section, the optimal ratio of cross-linkers will be used to study the effect of different weight percentages of montmorillonite on the compressive strength of the epoxy composite mortar.

**Fig. 1:**
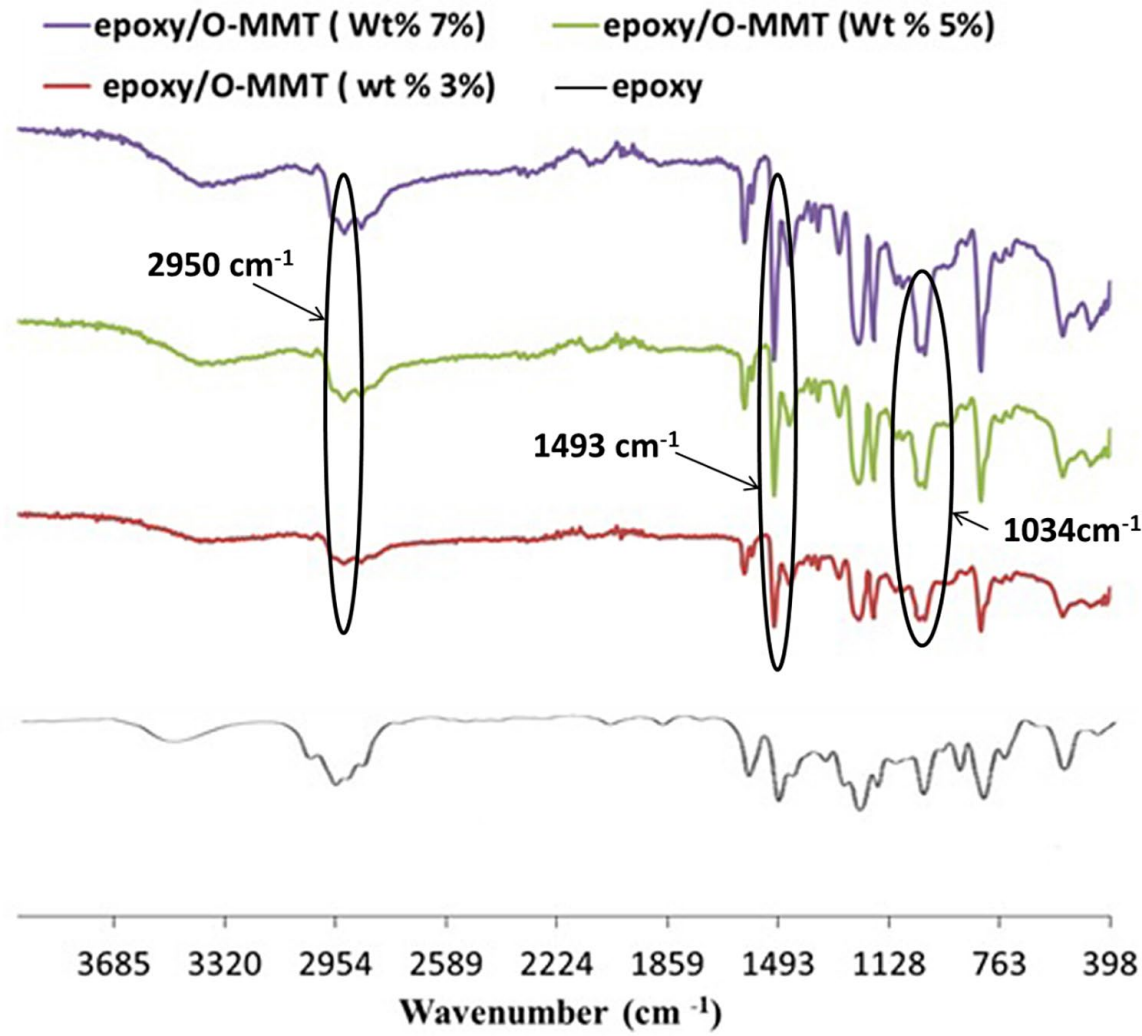
FT-IR charts of DGEBA resin cured with IPD: TETA / 75:25 ratio and its nanocomposites with weight% of O-MMT: 3%, 5%, and (d) 7%

Figure 1 shows the FT-IR charts of epoxy cured with IPD/TETA at a 75/25 ratio, both with and without reinforcement by organically modified montmorillonite (OMMT). In the FT-IR spectra of cured epoxy with OMMT, characteristic peaks were observed at 2950, 1034, and 1493 cm⁻¹, corresponding to the stretching of –CH₂, Si-O, and C–N groups, respectively, due to the presence of hexamine chains in the interlayer of MMT through cation exchange [21]. The presence of organic chains on MMT enhances the diffusion of OMMT and epoxy. The intensity of these peaks increases with the increasing weight% of OMMT in the epoxy/MMT composite.

### The effect of curing agent type on the gelation time of epoxy

**Fig. 2:**
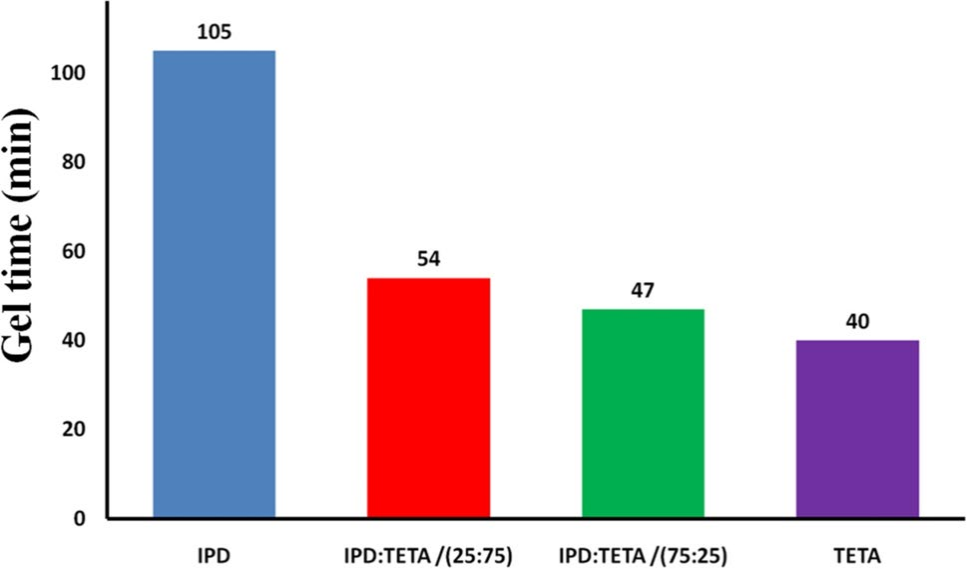
Measurements of Get time for curing of Diglycidyl ether of bisphenol-A epoxy using Isophoronediamine (IPD), Triethylenetetramine and different ratio IPD: TETA / 25:75 and 75:25

The gelation time of epoxy and its different composites was estimated as a function of the cross-linker used—either IPD, TETA, or a combination of IPD/TETA in different ratios (25/75 or 75/25), as illustrated in Fig. 2. The measured gel times were 105 min for epoxy cured with IPD and 40 min for epoxy cured with TETA. This difference can be attributed to TETA’s greater flexibility and lower steric hindrance compared to IPD, which is a cyclic hexane. The gel time decreased with an increasing amount of TETA, with the gel times for IPD/TETA at ratios of 25/75 and 75/25 being 54 and 47 min, respectively.

### The effect of curing agent type on compressive strength of epoxy mortar

**Fig. 3:**
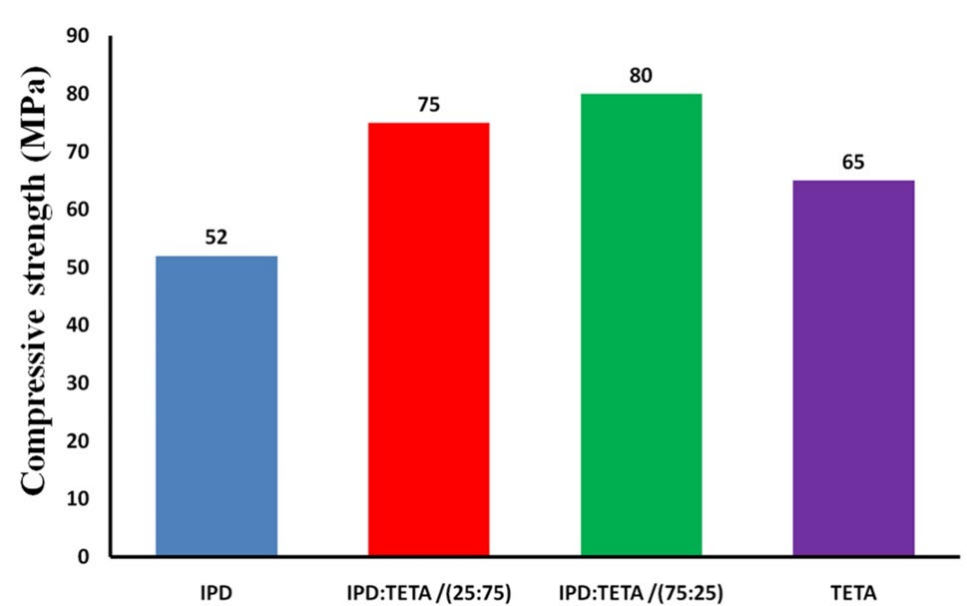
Measurements of compressive strength values of cured Diglycidyl ether of bisphenol-A epoxy by Isophoronediamine (IPD), Triethylenetetramine and different ratio IPD: TETA / 25:75 and 75:25

Various epoxy mortars were prepared with specific amounts, and cubic specimens were molded according to ASTM 579.01 Method B. The compressive strength values of epoxy cured with IPD and TETA were 52 MPa and 65 MPa, respectively, as shown in Fig. 3. TETA demonstrates better mechanical properties for epoxy compared to IPD, which can be attributed to TETA’s nature as an aliphatic linear cross-linker. Consequently, epoxy cured with TETA is less rigid than epoxy cured with IPD, a cycloaliphatic cross-linker. Different molar ratios of cross-linkers IPD/TETA (25/75 and 75/25) were used, and the compressive strength values for these mixtures were higher than those for individual cross-linkers, measuring 75 MPa and 80 MPa, respectively.

### The effect of reinforcing cured epoxy with organically modified montmorillonite (OMMT) was studied using epoxy cured with IPD/TETA at a molar ratio of 75/25

Epoxy resin cured with IPD/TETA at a molar ratio of 75/25, which exhibited the highest compressive strength, was chosen for reinforcement with different weight percentages of organically modified montmorillonite (OMMT), specifically 3%, 5%, and 7 wt%. In this section, the prepared composites of epoxy/IPD/TETA (75/25) with varying OMMT contents are characterized using various techniques.

### Morphology of the composites cured with a mixture of IPD and TETA

**Fig. 4:**
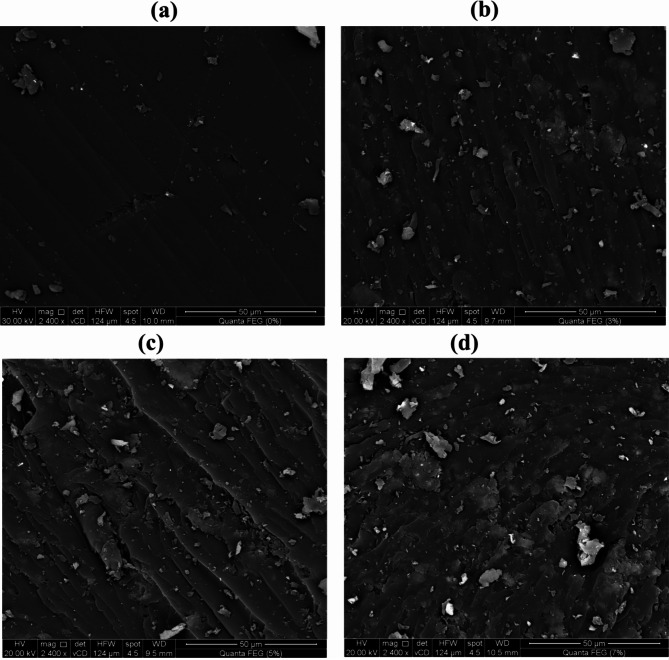
SEM micrographs of (**a**) cured Diglycidyl ether of bisphenol-A epoxy by IPD: TETA / 75:25 and its nanocomposites with weight% of O-MMT: (**b**) 3%, (**c**) 5% and (**d**) 7%

SEM image of the cured epoxy reveals a fractured surface with no signs of toughening, indicating a typical brittle fracture mode (Fig. 4a). In contrast, the fracture surfaces of cured epoxy with 3%, 5%, and 7% wt% organically modified montmorillonite (OMMT) are notably rougher (Fig. 4b and c, and 4d). The images show that the composites containing 5% and 7% OMMT have noticeable clay clusters that remain agglomerated in the epoxy matrix, whereas such clusters are minimal in the composite with 3% OMMT.

**Fig. 5:**
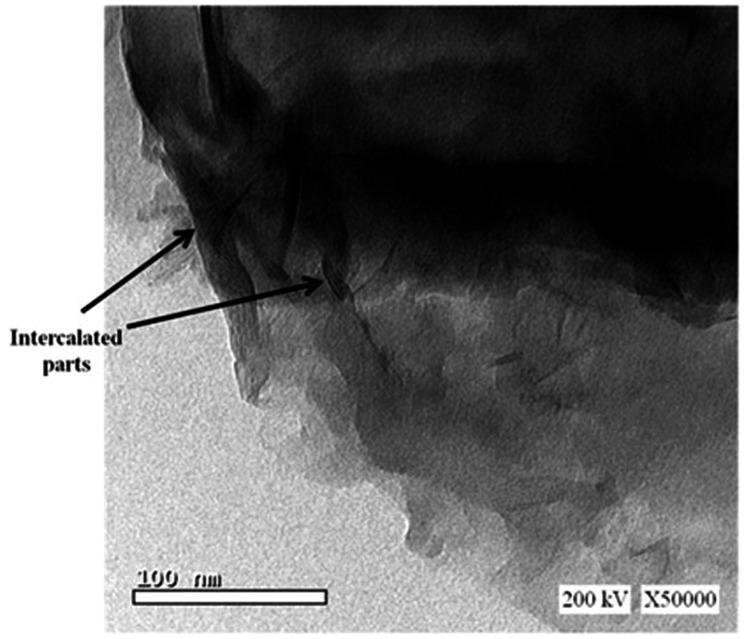
TEM image of cured Diglycidyl ether of bisphenol-A epoxy by IPD: TETA / 75:25 nanocomposite with 3 wt% of OMMT

The dispersion of clay within the epoxy composites plays a crucial role in enhancing the compressive strength of the cured epoxy. The composite with 3% OMMT demonstrates higher compressive strength compared to those with 5% and 7% OMMT. The dispersion of the epoxy composite with 3% OMMT was further investigated using TEM. As shown in Fig. 5, the TEM images reveal regular intercalation of the epoxy through the silicate layers, indicating effective insertion into the montmorillonite layers.

### Thermogravimetric analyses were conducted on epoxy cured with IPD: TETA / 75:25, as well as on various composites

**Fig. 6:**
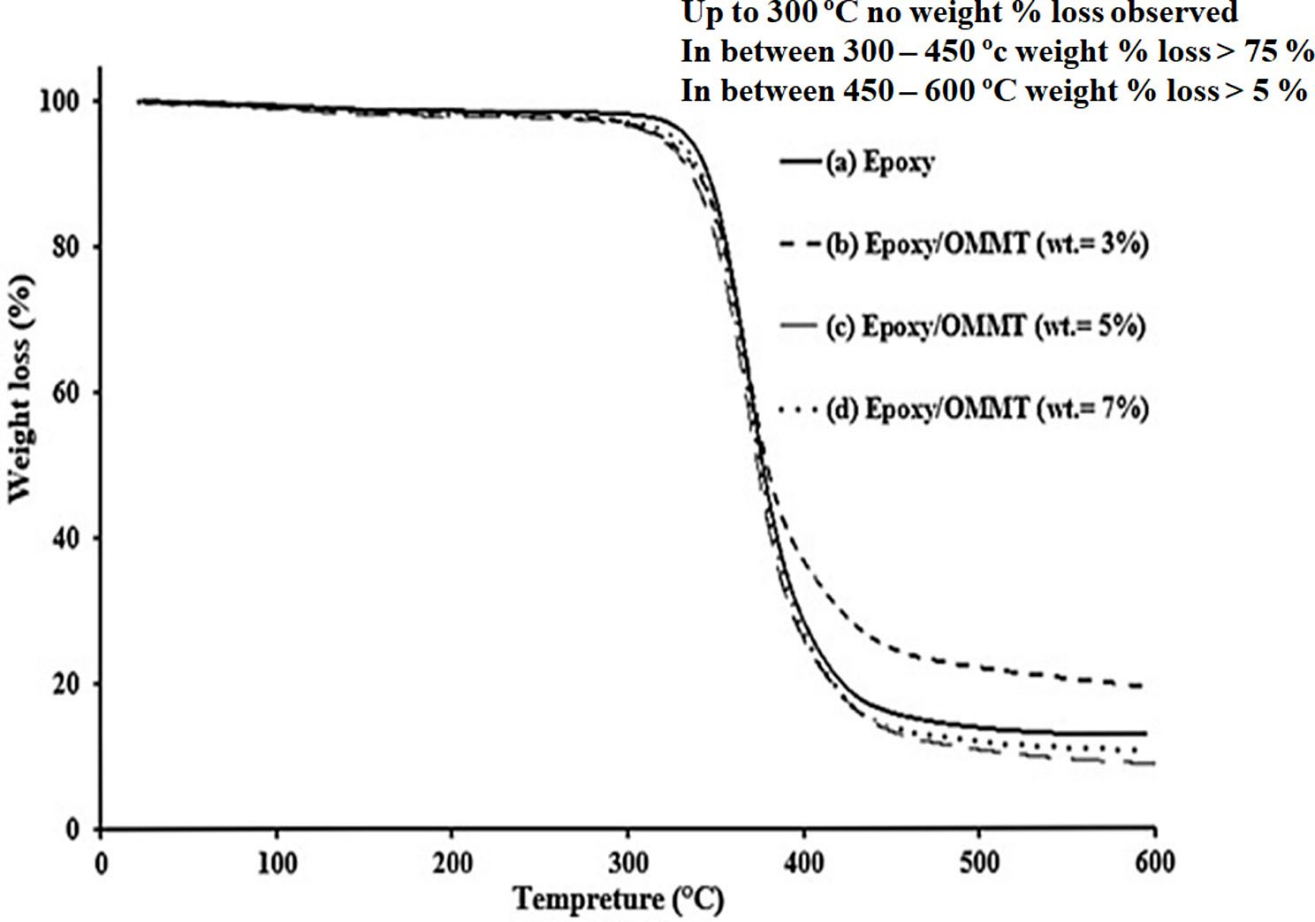
Thermogravimetric curves (a) cured Diglycidyl ether of bisphenol-A epoxy by IPD: TETA / 75:25 and its nanocomposites with weight% of O-MMT: (b) 3%, (c) 5% and (d) 7%

Figure 6 illustrates the thermogravimetric curves for pure epoxy and epoxy/clay composites with 3%, 5%, and 7% clay. There were no weight loss was observed up to 300 ºC. The major weight loss observed was found in two stages. In Stage one, major weight loss of pure epoxy and its nanocomposites about 80% occurred in temperature rang 300–450 ºC due to the degradation of epoxy chain to small volatiles molecules [27]. Which started at the initial decomposition temperature (IDT) of pure epoxy is 367.7 °C, while the initial decomposition temperatures for the epoxy nanocomposites containing 3%, 5%, and 7% OMMT are 367.7 °C, 370.7 °C, and 370 °C, respectively. In the second stage weight loss %, 450–600 ºC, pure epoxy epoxy nanocomposites containing 3%, 5%, and 7% OMMT are 10%, 12, 8, 10%. This indicates that the OMMT content has only a minor effect on the thermal behavior of the epoxy. However, the decomposition end temperature is slightly improved for the composite with 7% clay loading [28, 29].

### Compressive strength of epoxy mortars measurements

**Fig. 7:**
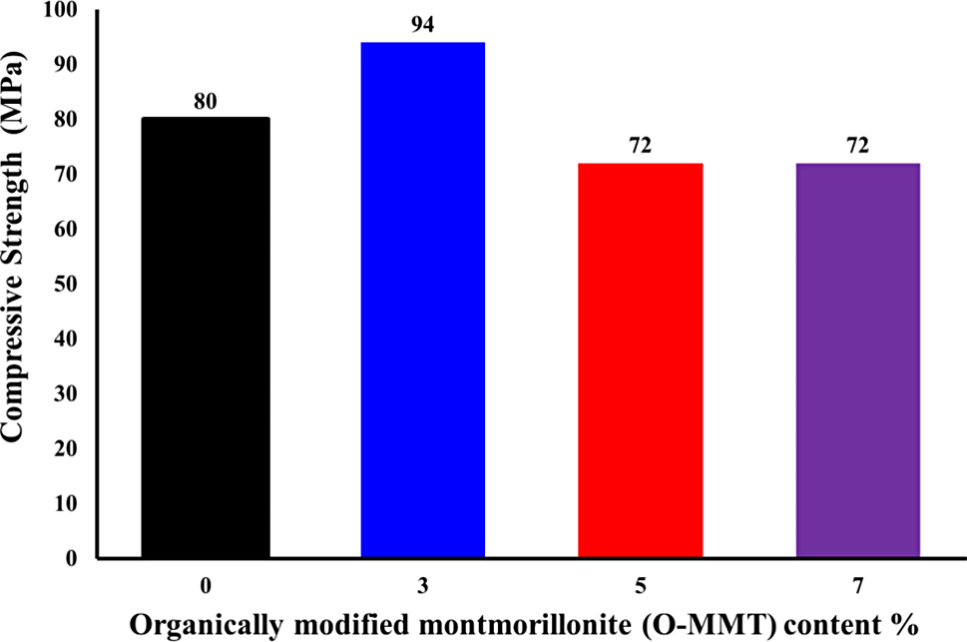
Compressive strength values as a function of content of (O-MMT) cured Diglycidyl ether of bisphenol-A epoxy by IPD: TETA / 75:25 and its nanocomposites with weight% of O-MMT: 3%, 5%, and 7%

Epoxy mortars, including both pure epoxy and epoxy/OMMT composites, were prepared in cubic shapes according to ASTM 579.01 Method B, using IPD: TETA / 75:25 as the curing agents, along with DGEBA resin and washed sand. The compressive strength of the epoxy was determined to be 80 MPa. The epoxy/OMMT composite containing 3 wt% OMMT has a compressive strength of 94 MPa. However, increasing the OMMT content to 7 wt% does not result in a significant improvement in the compressive strength of the epoxy (Fig. 7). This is consistent with the SEM results, which show that the full expansion of OMMT throughout the epoxy improves its compressive strength [30–32]. In comparison to the literature, the compressive strength of an epoxy/OMMT composite cured with IPD and containing 3 wt% OMMT is reported to be 72 MPa [25]. Using cross-linkers IPD/TETA in a molar ratio of 75/25 improved the compressive strength to 94 MPa.

## Conclusions

The diglycidyl ether of bisphenol-A epoxy/organically modified montmorillonite (DGEBA/OMMT) composite was prepared through in-situ polymerization using Isophorone diamine (IPD), Triethylenetetramine (TETA) and their different stoichiometric ratios. The optimal IPD/TETA ratio for achieving the highest compressive strength (94 MPa) of cured epoxy mortars was found to be 75/25. Using stoichiometric ratios of commercial cross-linkers for curing epoxy shows excellent compressive strength. Additionally, the compressive strength of the neat epoxy surpasses values reported in the literature. Cured epoxy composites reinforced with OMMT were characterized using Fourier-transform infrared spectroscopy (FT-IR), thermogravimetric analysis (TGA), transmission electron microscopy (TEM), and scanning electron microscopy (SEM). The presence of the organically modified clay was observed to reduce the gelation time of the epoxy resin. The thermal behavior of the epoxy/OMMT composites was consistent regardless of the OMMT weight%. SEM and X-ray diffraction (XRD) analyses demonstrated effective expansion of OMMT at 3 wt% loading within the epoxy matrix. The DGEBA/OMMT composites cured with IPD/TETA (75/25) and containing 3 wt% OMMT exhibited a compressive strength is equal 94 MPa which approximately 31% higher compared to literature values for epoxy cured with IPD alone at the same OMMT weight%.

## Data Availability

No datasets were generated or analysed during the current study.

## Abbreviations

DGEBA: Diglycidyl ether bisphenol-A
IPD: Isophorenediamine
TETA: Triethylenetetramine
OMMT: organically modified montmorillonite
